# Stop the bleed campaign: A qualitative study from our experience from the middle east

**DOI:** 10.1016/j.amsu.2018.10.013

**Published:** 2018-10-24

**Authors:** Salman AlSabah, Eliana Al Haddad, Fahd AlSaleh

**Affiliations:** a Kuwait University, Mubarak Al-Kabeer Hospital, Kuwait City, Kuwait; b Al Amiri Hospital, Mubarak Al-Kabeer Hospital, Kuwait City, Kuwait; c Mubarak Al-Kabeer Hospital, Kuwait

**Keywords:** First aid, Bystander, Basic life support, Campaign, Stop the bleed

## Abstract

**Background:**

Bleeding due to unintentional injuries are a leading cause of death in the younger population. The immediate involvement of lay bystanders has been proven to be imperative in outcomes, however, there still is less than 30% of out-of-hospital resuscitation attempts initiated by them.

**Study design:**

The Stop the Bleed campaign was initiated in Kuwait in September-2017, with the aim to raise awareness and train the general public on emergency situations. A survey questionnaire was distributed to a sample of 150 participants to assess their comprehension.

**Results:**

A total of 1531 participants were trained by the campaign. More than half of the participants have had no previous training of any sort for emergency situations, with the majority (86%) of those queered expressing desire to learn about how to deal with trauma and bleeding cases. After training, most participants were able to demonstrate knowledge of how to deal with unstoppable bleeding, know where and when to place a tourniquet, knew how to respond to epistaxis, and the ability to recognize signs of internal bleeding, with 89% expressing that the ‘Stop the Bleed’ campaign was useful for promoting health and raising awareness on safety of individuals.

**Conclusion:**

With the appropriate first-aid training and skill retention, lay members of the public can potentially contribute to a positive and important post-trauma medical response.

## Highlights

• Bleeding due to unintentional injuries are a leading cause of death in the younger population.
• Immediate involvement of bystanders is imperative in outcomes, however, <30% of resuscitation attempts are initiated.
• The Stop the Bleed campaign was initiated in Kuwait to raise awareness and train the general public on emergency situations.
• In our paper, we aimed to assess the effectiveness of this campaign.

## 1 Introduction

Unintentional injuries are the leading cause of death among those that are 1–44 years of age in the United States, resulting in approximately 2.6 million hospitalizations, 34.9 million emergency room visits and 87.6 million medical office visits per year [1]. With the introduction of cardiopulmonary resuscitation (CPR) and basic life support (BLS) in the 1960's [2], these numbers have indeed improved significantly, however, when it came to looking at the involvement of the general public in assisting with injuries, there still is less than 30% of out-of-hospital resuscitation attempts initiated by lay bystanders.

Basic first aid training has been shown to prepare bystanders to react and provide immediate and efficient treatment for a wide variety of incidents. These could range from injuries acquired in a home setting, to mass casualty events such as car crashes and natural disasters. Such training would include alerting the emergency medical system, maintaining the airway, breathing & circulation, respiratory & cardiac arrest, and hemorrhage control. However, even though the response time in emergency situations is critical, the first aid provided must be performed properly in order to prevent further complications and potentially save lives [3].

The 'Stop the Bleed' campaign was initiated by a federal interagency workgroup convened by the National Security Council Staff of The White House in 2016 with the purpose of building national resilience by better preparing the public to save lives by raising awareness of basic actions to stop life threatening bleeding following everyday emergencies and man-made and natural disasters [4]. It has recently been adapted in Kuwait, with the campaign launching there in September 2017 by the Kuwait Surgeons Association in partnership with the Health Promotion Department of the ministry of health (MOH).

The objectives of the campaign are 1) To raise awareness among the gulf adult population on how to stop bleeding occurring due to trauma, 2) to assess the current knowledge the gulf adult population have in regard to Stop the bleed 3) to find out if the gulf adult population feel the need for training courses on first aid skills 4) to find new avenues to better promote information sources for the public regarding first aid knowledge. In this paper, we report on the outcomes and success of the campaign thus far.

## 2 Methods

The first part of the campaign began in September 2017 and involved the recruitment and training of medical professionals on how to teach first aid and basic life support training. Training kits were provided for this education process.

Part of the campaign was also training medical students at the faculty of medicine in Kuwait University. Fifth year medical students were given a course on bleeding control and were assessed before and after the course accordingly.

After which, on February 1st, 2018, the campaign was launched to the general public at one of the most popular locations in Kuwait. There, the public were provided with courses in which they were trained on how to deal with emergency situations as first responders.

Along side that, public marketing videos were filmed and displayed on local TV channels, as well as spread via social media platforms for the public.

A survey questionnaire composed of 20 questions was distributed to a sample of 150 participants, for which they were asked about socio-demographic data including age, gender, nationality, education level & occupation; History of trauma/injury, training on emergency rescue received before, information sources on treatment of bleeding cases, emergency phone number knowledge and questions regarding on how to stop bleeding during different trauma situations. The initial data was tabulated and the statistical analysis of the data was carried out using SPSS software version 22.

The work is fully compliant with the Standards for Reporting Qualitative Research [5].

## 3 Results

A total of 1531 participants were trained by the campaign that began on February 1st, 2018. The survey previously mentioned was distributed to 150 randomly chosen participants, for which they were queered about multiple aspects related to trauma. 50% of the randomly chosen participants were female, with the majority (44%) of all participants ranging in age between 21 and 30 years old. More than 45% of the participants queered were found to have a university degree (Table 1).

**Table 1:** Demographics of participants.

N = 150	Percentage
Gender (F)	50%
Age 21–30 31–40	44% 34%
Level of education High school diploma University graduate	40% 45.3%

When it came to looking at previous knowledge of first aid, it was seen that more than half of the participants have had no previous training of any sort for emergency situations, while 55.3% of the participants expressed that the knowledge that they have gained about first aid came from first aid courses they have participated in at some point, with the majority (86%) of those queered expressing that they have a want to learn about how to deal with trauma and bleeding cases.

When asked about their first aid knowledge after going through the Stop the Bleed training campaign, most participants were able to demonstrate knowledge of how to deal with unstoppable bleeding, know where and when to place a tourniquet, knew how to respond to epistaxis, as well as able to recognize signs of internal bleeding. 89% of them expressed that they felt that the ‘Stop the Bleed’ campaign was useful for promoting health and raising awareness on safety of individuals (Table 2).

**Table 2:** Results from the survey.

N = 150	Percentage
History of Trauma (personal or relative)	48.7%
Training for Emergency None Some form of training	56.7% 39%
Main sources of info for first aid treatment Internet/social media First aid courses Physicians	50.7% 55.3% 20.7%
Expressed want to learn trauma and bleeding first aid skills Reason to refrain from helping accident victims Fear of endangering the victim Medico-legal responsibility	**86%** 50% 50%
How to deal with bleeding Knew how to help a trauma victim with unstoppable bleeding knew what to do in case on unstoppable bleeding with the dressing soaked in blood Did not know how to respond	42.7% 45% 31%
Knew where the tourniquet must be applied in relation to a bleeding site	84%
How to respond to epistaxis knew the best position to place the head in case of epistaxis knew when a person with epistaxis had to be transferred to a medical center	32% 70%
Knew signs of internal bleeding	64%
**Felt that “Stop the Bleed” campaign is useful for promoting health & raising awareness on safety of individuals**	**89%**

**Fig. 1:**
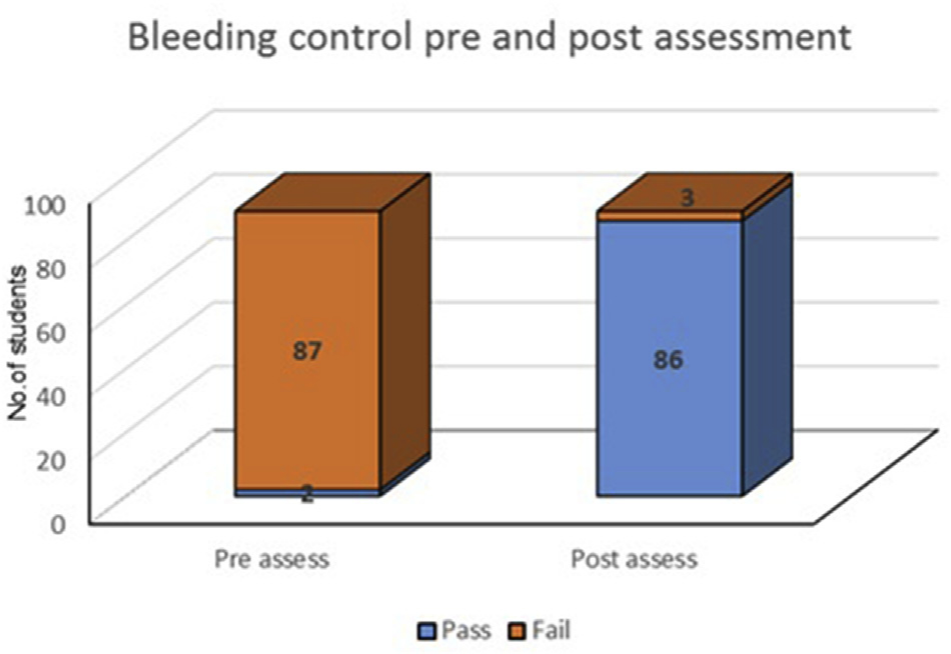
Results from the Assessment Pre and Post-delivery of the Bleeding Control Course.

Fifth year medical students were assessed pre and post delivering a bleeding control course at the faculty of medicine. With a passing grade of 70%, before delivering the course, only 2% of the students passed the assessment, while that number showed a drastic increase to a 96% pass rate after the course was delivered (Fig. 1).

## 4 Discussion

First aid training is now considered an important skill to gain for most people around the world, with over 50% of adults having had received some form of training at some point in their lives [[6], [7], [8]]. With Kuwait being situated in the gulf region, it is considered as being surrounded by countries constantly plagued by war and misfortune. Nevertheless, up until recently, Kuwait has been mostly spared from the volume and impact of terrorist attacks seen in other countries around it, and therefore, the mosque bombing that occurred in 2015 that claimed the lives of 27 people came as a shock. The occurrence of this incident was a wake-up call, highlighting the lack of first-aid training and response to disasters in the country, and as further proven by our results, 57% of the population queered by our survey have had no prior exposure to any training of this sort, with 86% of them actually expressing an interest in learning about this topic, and therefore, the Stop the Bleed campaign was initiated.

Unintentional injuries are the leading cause of death among those that are 1–44 years of age in the USA [9]. With a population of approximately 4 million people in Kuwait, it has been documented that the most common cause of death in patients younger than 40 was that due to trauma from road traffic accidents, and accounted for 1.6 deaths per day. In a study conducted by Larsson et al. [6] on a Swedish population, they were able to demonstrate that 14% of the people involved reported being present at the scene of a road traffic accident in the five years prior to the investigation. According to the same study, a bystander at a crash site often concludes that first aid is unnecessary, with the rate of immediate action being less than 35% on victims as reported by ambulance persona arriving on the scene [[10], [11], [12], [13]]. Furthermore, earlier studies were also able to demonstrate that professionals often were of the opinion that bystanders could have done more [14], with other studies showing that first-aid training predicted actual utilization of these first-aid skills [15]. This data combined is significant enough to emphasize the importance of extensive first-aid training of the general public. The results of the campaign that we initiated showed a significant improvement in the type of response of the general public to basic traumas assessed, proving greater perceived abilities to handle a medical emergency by those that took part in it. This further stresses the importance of conducting such campaigns.

While it may seem self-explanatory that public education on this topic should begin with health professionals, it has been shown that many of them however have not acquired life-supporting first aid skills [16,17]. Medical students [18,19], nurses [20], and other health professionals [18] have been shown to have retention levels equally as poor as bystander lay groups. Providing the bleeding control course to our fifth year medical students was able to stress the importance of such training, demonstrating a huge advancement in the knowledge of the medical students on bleeding control. As seen from our results, the majority of persons that participated in the campaign were those of younger ages, as well as having received at least a high-school education. This is consistent with results obtained from previous studies, which showed a correlation between first-aid and CPR-training with younger age and a higher level of education [6,7]. These findings may be related to a generally more positive attitude towards education in those groups of participants. This also helps in directing the focus of attention as to whom and where to provide this training, possibly conducting this campaign on university campuses around the country. However, all in all, nearly 90% of those that participated in our study felt that the Stop the Bleed campaign was useful for promoting health and raising awareness on the safety of individuals.

The limitations of this study include the fact that first-aid ability was measured using self-reports, which may not accurately reflect practical competence. Furthermore, even though we attempted to make as random of a selection as we could have to assess this topic, the sample of 150 randomly chosen individuals of the 1531 participants may not accurately reflect the responses of all those that took part in the campaign, as well as possibly predisposing to selection bias. However, using self-reports allowed us to obtain a larger sample at lower cost than would be expected from assessing practical competence.

## 5 Conclusion

First-aid training can increase both expected and actual utilization of first-aid skills as well as perceived competence in implementing those skills. With the appropriate training and skill retention, lay members of the public can potentially contribute to a post-trauma medical response.

## Provenance and peer review

Not commissioned, externally peer reviewed.

## Ethical approval

Ethical approval to conduct the study was obtained from the Ministry of Health and Kuwait Institute for Medical Specialization Ethical Approval Board.

## Sources of funding

None.

## Author contribution

Dr.Salman AlSabah: study design, data collection.

Dr.Eliana Al Haddad: data collection, data analysis, writing, editing.

Dr. Fahd AlSaleh: writing, editing.

## Conflicts of interest

None.

## Research registry number

researchregistry3597.

## Guarantor

Dr. Salman AlSabah.
